# Cross-Talk between TLR4 and FcγReceptorIII (CD16) Pathways

**DOI:** 10.1371/journal.ppat.1000464

**Published:** 2009-06-05

**Authors:** Daniel Rittirsch, Michael A. Flierl, Danielle E. Day, Brian A. Nadeau, Firas S. Zetoune, J. Vidya Sarma, Clement M. Werner, Guido A. Wanner, Hans-Peter Simmen, Markus S. Huber-Lang, Peter A. Ward

**Affiliations:** 1 Department of Pathology, University of Michigan Medical School, Ann Arbor, Michigan, United States of America; 2 Department of Traumatology, University Hospital Zurich, Zurich, Switzerland; 3 Department of Traumatology, Hand-, Plastic-, and Reconstructive Surgery, University Hospital Ulm, Ulm, Germany; NIH/NIAID, United States of America

## Abstract

Pathogen-pattern-recognition by Toll-like receptors (TLRs) and pathogen clearance after immune complex formation via engagement with Fc receptors (FcRs) represent central mechanisms that trigger the immune and inflammatory responses. In the present study, a linkage between TLR4 and FcγR was evaluated *in vitro* and *in vivo*. Most strikingly, *in vitro* activation of phagocytes by IgG immune complexes (IgGIC) resulted in an association of TLR4 with FcγRIII (CD16) based on co-immunoprecipitation analyses. Neutrophils and macrophages from TLR4 mutant (mut) mice were unresponsive to either lipopolysaccharide (LPS) or IgGIC *in vitro*, as determined by cytokine production. This phenomenon was accompanied by the inability to phosphorylate tyrosine residues within immunoreceptor tyrosine-based activation motifs (ITAMs) of the FcRγ-subunit. To transfer these findings *in vivo*, two different models of acute lung injury (ALI) induced by intratracheal administration of either LPS or IgGIC were employed. As expected, LPS-induced ALI was abolished in TLR4 mut and TLR4^−/−^ mice. Unexpectedly, TLR4 mut and TLR4^−/−^ mice were also resistant to development of ALI following IgGIC deposition in the lungs. In conclusion, our findings suggest that TLR4 and FcγRIII pathways are structurally and functionally connected at the receptor level and that TLR4 is indispensable for FcγRIII signaling via FcRγ-subunit activation.

## Author Summary

The immune system is traditionally divided into innate and adaptive entities. Pattern-recognition receptors (PRRs) collectively recognize molecular structures of invading microorganisms, followed by initiation of immune responses. PRRs comprise the toll-like receptor (TLR) family, including TLR4, which is essential for responses to bacterial lipopolysaccharide (LPS). As part of the adaptive immune system, Fc receptors (FcRs) on immune cells recognize antigen–antibody complexes and link antibody-mediated immune responses to cellular effector functions. Here, we describe cross-talk between the pathogen-recognition-receptor toll-like receptor 4 (TLR4) and receptors for IgG immune complexes (IgGIC), Fcγ receptors (FcγRs). We found that TLR4 is involved in FcγRIII (CD16) signaling and that heterodimerization of TLR4 and FcγRIII occurs in the presence of IgGIC but not LPS. Consequently, dysfunctional TLR4 signaling results in unresponsiveness of immune cells *in vitro* to both LPS and IgGIC, resulting in absence of acute lung injury after intratracheal administration of LPS or intrapulmonary immune complex deposition. In summary, we describe that TLR4 and FcγRIII pathways are structurally and functionally connected. These findings provide new insights of the interplay between innate and adaptive immunity, which closely interact with each other at the receptor level and post receptor signaling pathways.

## Introduction

The immune system is traditionally divided into innate and adaptive entities. Adaptive immunity is organized around T cells and B cells and requires a process of maturation and clonal selection of lymphocytes. In contrast, innate immunity can be immediately activated during the onset of infection in order to control replication of pathogenic microbes and bring about their clearance from tissues or blood. As an important aspect of innate immunity, pattern-recognition receptors (PRRs) collectively recognize lipid, carbohydrate, peptide, and nucleic-acid structures of invading microorganisms [1]. PRRs comprise the toll-like receptor family (TLR), which consists of at least 12 different evolutionarily conserved membrane proteins that trigger innate immune responses [2]. Initially identified in 1997, TLR4 represents the most thoroughly investigated TLR [3]. TLR4 is essential for responses to bacterial lipopolysaccharide (LPS), a well-known pathogen-associated molecular pattern (PAMP) [3],[4]. Besides LPS, various endogenous ligands, such as hyaluronan and high mobility group box 1 protein (HMGB1), appear to engage TLR4 [5],[6]. After binding of LPS to the TLR4/MD-2/CD14 receptor complex, activation of the intracellular signaling pathway is initiated, ultimately leading to NF-κB activation and its translocation to the nucleus, resulting in subsequent cytokine/chemokine production and release [7].

As part of the adaptive immune system, antibodies of high affinity binding specifically recognize and neutralize intruding pathogens or their products. After antibody binding to antigen, the Fc domain of immunoglobulin (Ig) is recognized by Fc receptors (FcRs) which are predominantly expressed on immune and inflammatory cells and thereby link antibody-mediated (humoral) immune responses to cellular effector functions [8],[9]. Specific FcRs exist for all classes of immunoglobulins. Binding of IgGs to FcγRs on phagocytes triggers a wide variety of cellular functions including phagocytosis, release of inflammatory mediators, and clearance of immune complexes [8]. FcγRs specifically bind IgG and are divided into four subclasses. FcγRI (CD64), FcγRIII (CD16), and FcγRIV are activating receptors, while FcγRII (CD32) mediates inhibitory functions. The cellular response is determined by the balance between activating (ITAM, immunoreceptor tyrosine-based activation motif) and inhibitory (ITIM, immunoreceptor tyrosine-based inhibitory motif) signals [10],[11],[12],[13].

Despite extensive research in the past, the highly complex regulation of innate and adaptive immunity and their interactions are still poorly understood. It has been suggested that adaptive immune responses are controlled by innate immune recognition and vice versa [14],[15],[16]. In particular, TLRs and FcγRs are considered to be important regulators of immune responses [13],[17]. Recently, evidence has emerged that there is indirect interaction between TLR4 and FcγR pathways. TLR4 has been shown to up-regulate FcγR expression in experimental immune complex arthritis; inhibition of TLR4 resulted in attenuation of *in vivo* cytokine release in models of glomerulonephritis and rheumatoid arthritis [18],[19],[20]. In the present study, we addressed the question as to whether there is a direct link between TLR4 and FcγR pathways *in vitro* and *in vivo*.

## Results

### Exclusion of LPS Contamination of Reagents

In the past, the investigation of TLR4 faced the problem of LPS contamination, which imposed considerable restrictions on the interpretation of data [5]. Therefore, the LPS concentration was determined in reagents used for lung injury induction by deposition of IgG immune complexes (IgGIC), such as DPBS, anti-BSA IgG and BSA, although none of these reagents had been prepared using bacterial (*E.coli*) systems. Using Limulus Amebocyte Lysate Kinetic-QCL assay, LPS levels were not detectable (<5×10^−3^ units/ml) in any of the reagents (data not shown), suggesting that *in vitro* stimulation by IgGIC is based upon a genuine agonist effect that is not due to LPS contamination. In addition to determination of LPS contamination (see above), DPBS, anti-BSA IgG and BSA were subjected to endotoxin removal by solid-phase polymyxin. Using the polymyxin-treated reagents, immune complexes were generated and then applied in *in vitro* experiments or the reagents were administered in mice for the formation of immune complexes *in vivo*. Furthermore, commercially available, preformed peroxidase/anti-peroxidase immune complexes (PAP IgGIC) were used at the same concentration in order to confirm the results using BSA IgGIC or polymyxin-treated BSA IgGIC. The results of both, polymyxin-treated BSA IgGIC and PAP IgGIC, are presented in the corresponding figures. In summary, using different *in vitro* and *in vivo* approaches, it is highly unlikely that any of the effects following IgGIC stimulation in the present study are based on LPS contamination of the reagents.

### Association between TLR4 and FcγRIII after IgG Immune Complex Activation

**Figure 1:**
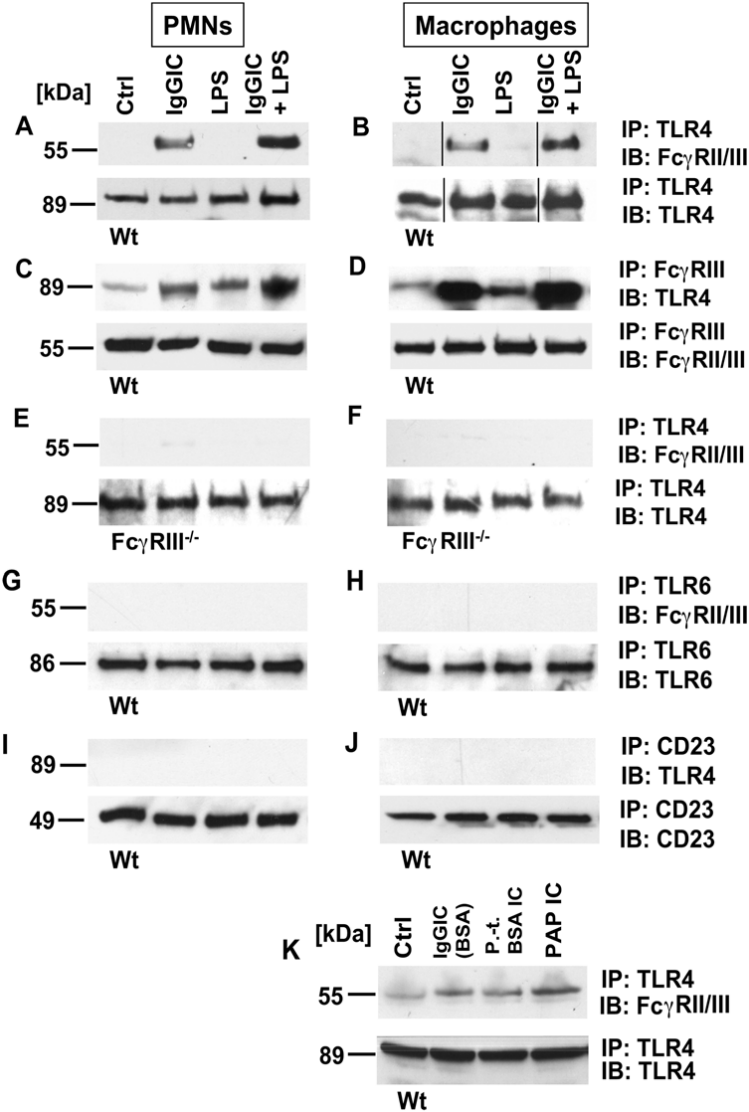
Association between TLR4 and FcRγIII. Peritoneal PMNs and macrophages (3×10^6^ cells/ml) from Wt mice and FcRγ-subunit^−/−^ mice were incubated *in vitro* for 30 min with either IgG immune complexes (IgGIC; 100 µg/ml), LPS (20 ng/ml), or the combination. (A,B) Western blot analysis (IB) for FcγRIII of Wt PMN or macrophage lysates co-immunoprecipitated (IP) with anti-TLR4. (C,D) Reverse direction immunoprecipitation using anti-FcγRII/III IgG followed by Western blot analysis for TLR4. (E,F) Western blot analysis for FcγRIII of PMNs or macrophages from FcγRIII^−/−^ co-immunoprecipitated (IP) with anti-TLR4. (G,H) Samples were immunoprecipitated with anti-TLR6 IgG and probed for FcγRIII. (I,J) Immunoprecipitation with anti-CD23 followed by Western blots using anti-TLR4 IgG. (K) Western blots (IB) of cell lysates of Wt macrophages that were incubated for 30 min with BSA IgG immune complexes (IgGIC; 100 µg/ml), polymyxin-treated BSA IgG immune complexes (p.-t. BSA IC; 100 µg/ml) or peroxidase/anti-peroxidase IgGIC immune complexes (PAP IC, 100 µg/ml). IB for FcγRIII of Wt macrophage lysates co-immunoprecipitated (IP) with anti-TLR4. Corresponding loading controls are displayed in lower panels.

In order to assess whether crosstalk between TLR4 and FcγR might occur at the receptor level, neutrophils (PMNs) and macrophages from wild-type (Wt) mice were incubated *in vitro* with IgGIC, LPS, or the combination of the two. After incubation, cell lysates were immunoprecipitated (IP) with anti-TLR4 and then analyzed for FcγRII/III by immunoblotting (IB). As shown in Figure 1A,B, immunoprecipitated TLR4 was associated with FcγR after cell exposure to IgGIC. Inversely, LPS incubation did not result in an association of both receptors as indicated by the absence of bands for FcγR, whereas the combination of LPS+IgGIC seemed to enhance the signal for FcγR co-immunoprecipitated by anti-TLR4 IgG (Figure 1A,B). The band for FcγR under the conditions described above indicated a protein mass of 55 kDa, in accord with the reported molecular weight for FcγRIII [21],[22]. In contrast, there was no band at the 40 kDa position (data not shown), the molecular weight of FcγRII, which is also recognized by the anti-FcγR antibody (mAb, clone 2.4G2) used for Western blot analyses [23],[24]. In accord with Figure 1A,B, reverse direction immunoprecipitation using FcγRIII antibody followed TLR4 Western blots revealed bands at around 90 kDa, consistent with the reported molecular weight of TLR4 (Figure 1C,D). However, under these conditions bands also occurred after stimulation of phagocytes with LPS (Figure 1C,D), which may suggest that FcγRIII and TLR4 heterodimerize upon LPS stimulation, although to a lesser extent as compared to IgGIC treated cells. When PMNs and macrophages from FcγRIII^−/−^ mice were exposed to the same *in vitro* conditions (IgGIC, LPS, LPS+IgGIC), the band for FcγRIII failed to appear, confirming its specificity (Figure 1E,F). In order to examine whether the interaction between TLR4 and FcγRIII was specific for these two receptors or whether there also might be multimerization with other TLRs or Fc receptors, lysates from Wt phagocytic cells under various conditions (see above) were subjected to immunoprecipitation with anti-TLR6 or anti-CD23 (anti-FcεRII), followed by Western blots for FcγRIII or TLR4, respectively (Figure 1G–J). In both combinations, specific bands for either FcγRIII (after immunoprecipitation with anti-TLR6; Figure 1G,H) or TLR4 (immunoprecipitation of cell lysates with anti-TLR6; Figure 1I,J) failed to appear, whereas the strong bands in the lower panels (loading controls) demonstrate that immunoprecipitation of the samples worked properly. In addition, macrophages from Wt mice were incubated with polymyxin-treated BSA IgGIC and PAP IgGIC, followed by immunoprecipitation with anti-TLR4 and Western blotting with anti-FcγRIII. As shown in Figure 1K, receptor heterodimerization occurred under these conditions as well, confirming the results shown in Figure 1A,B.

In summary, these findings indicate that association of TLR4 and FcγRIII occurs following activation of phagocytes with IgGIC and/or LPS and that this receptor association is a specific phenomenon for FcγRIII and TLR4.

### Attenuated *In Vitro* Cytokine Production by TLR4 Mutant PMNs and Macrophages Following IgGIC or LPS Exposure

**Figure 2:**
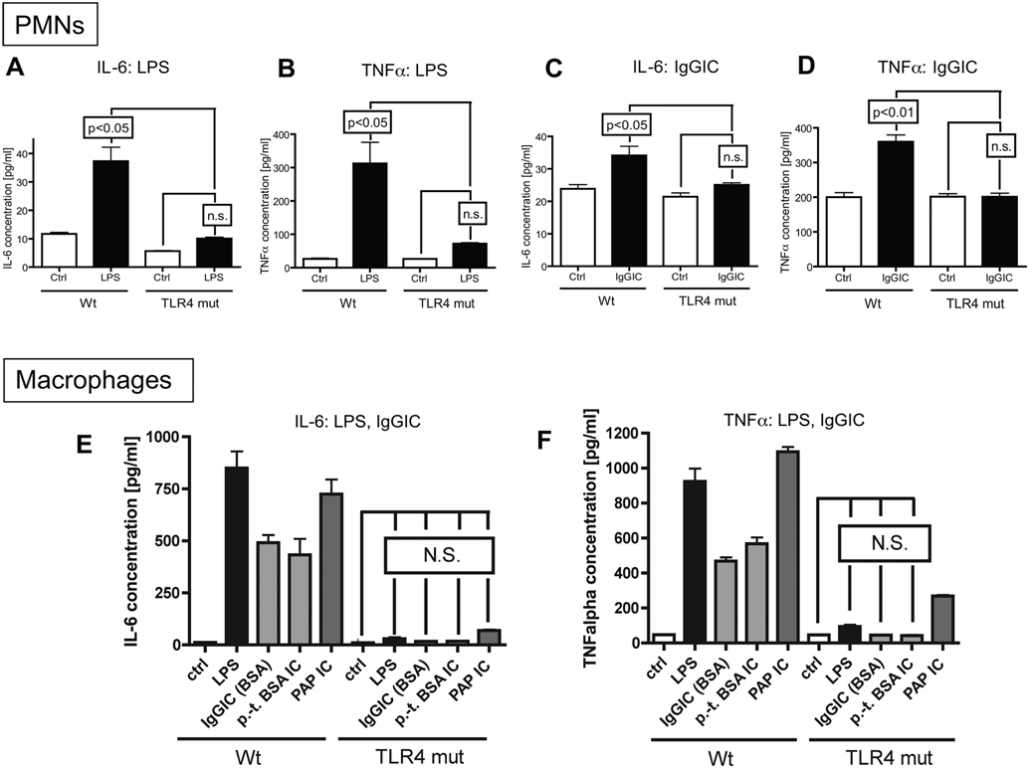
*In vitro* cytokine responses of elicited peritoneal PMNs and macrophages to LPS and IgGIC. *In vitro* cytokine responses of elicited peritoneal PMNs (A–D) and macrophages (E,F). Cells (3×10^6^ cells/ml) from either Wt or TLR4 mut mice were incubated for 4 hr with LPS (20 ng/ml) or IgGIC; 100 µg/ml), respectively. In addition, macrophages were incubated with polymyxin-treated BSA IgG immune complexes (p.-t. BSA IC, 100 µg/ml) or peroxidase/anti-peroxidase IgGIC immune complexes (PAP IC, 100 µg/ml). (A) IL-6 release from PMNs after LPS stimulation. (B) TNFα levels after incubation of PMNs with LPS. (C) Concentration of IL-6 in supernatants when PMNs were exposed to IgGIC. (D) Production of TNFα by PMNs and macrophages in the presence of IgGIC. Ctrl = control levels of non-stimulated cells. (E) Release of IL-6 by macrophages into supernatant fluids after stimulation with LPS, IgGIC, p.-t. BSA IC, or PAP IC. (F) TNFα production by macrophages exposed to LPS, IgGIC, p.-t. BSA IC, or PAP IC. The experiments were performed in triplicates for each condition (each bar) with n≥3 donors of cells for each mouse strain, Wt or TLR4 mut. Differences between controls and stimulated cells were—if not otherwise noted—statistically significant (p<0.05).

Elicited peritoneal neutrophils (PMNs) and macrophages were obtained from Wt and TLR4 mut mice. The cells were incubated *in vitro* with IgGIC or LPS. Subsequently, supernatant fluids were collected and evaluated by ELISA for intereukin-6 (IL-6) and tumour necrosis factor alpha (TNFα) levels (Figure 2). PMNs from Wt mice showed significant release of IL-6 and TNFα after exposure to either IgGIC or LPS. In the case of TLR4 mut PMNs, cytokine responses to IgGIC or LPS were lost (Figure 2A–D). When peritoneal macrophages were employed in the same protocol, similar results were found (Figure 2E,F). There was a 4-fold increase in IL-6 after exposure of Wt macrophages to LPS, and a 3-fold increase in IL-6 after IgGIC exposure (Figure 2E). Likewise, there was a robust release of TNFα by Wt macrophages into supernatant fluids after stimulation with IgGIC or LPS. When TLR4 mut macrophages were used under the same conditions, IL-6 and TNFα responses to IgGIC or LPS were greatly abolished (Figure 2E,F). Similar results were found when macrophages were incubated with polymyxin-treated BSA IgGIC or PAP IgGIC indicating that the results are reproducible and not based on LPS contamination of the reagents (Figure 2E,F). Thus, the lack of a functional TLR4 is associated with the *in vitro* inability of PMNs and macrophages to respond to LPS or IgGIC.

In order to assess if the impaired response of TLR4 mut cells observed *in vitro* might be due to a general impairment of the inflammatory response, peritoneal PMNs and macrophages from Wt and TLR4 mut mice were exposed to opsonized zymosan particles as well as to Pam3Cys, which is a specific ligand for TLR2 [25],[26],[27] . As displayed in Figure S1, Wt cells showed a significant increase of IL-6 (Figure S1A,C,E,G) and TNFα (Figure S1B,D,F,H) release when incubated *in vitro* with Pam3Cys or opsonized zymosan particles. In contrast to the findings described above (incubation with LPS or IgGIC), PMNs (Figure S1A–D) and macrophages (Figure S1E–H) from TLR4 mut mice showed full responses for IL-6 and TNFα when incubated with opsonized zymosan particles or Pam3Cys. These data indicate that the ability to produce cytokines in response to non-TLR4 agonists is intact in TLR4 mut cells and that the impairment of the inflammatory response to LPS and IgGIC is specific for the non-functional TLR4 protein.

**Figure 3:**
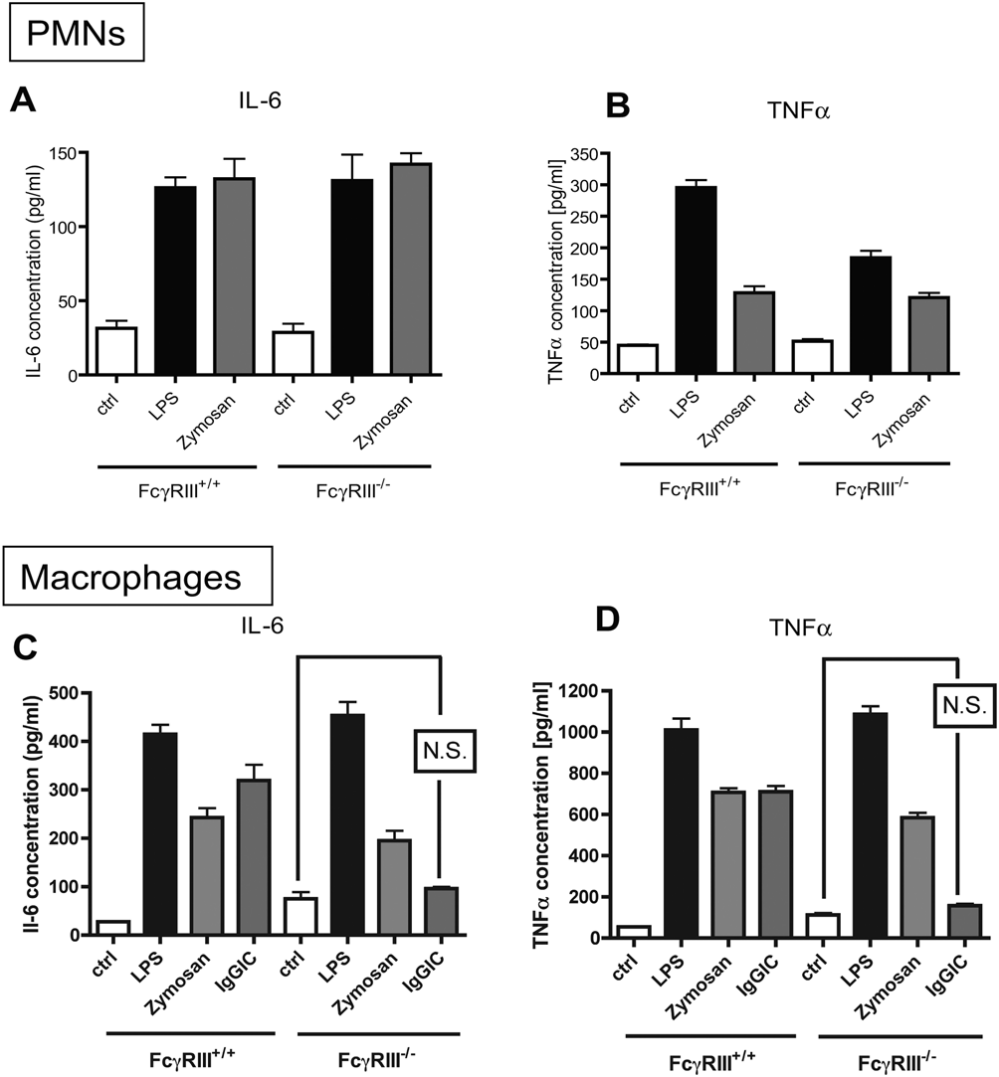
Responsiveness of FcγRIII-deficient phagocytes to LPS. Peritoneal PMNs (A,B) and macrophages (C,D) from Wt and FcγRIII^−/−^ mice were incubated to LPS (100 ng/ml) or Zymosan (300 µg/ml), or IgG immune complexes (IgGIC; 100 µg/ml; macrophages only), and supernatant fluids were analyzed for IL-6 and TNFα levels. Ctrl = control levels of non-stimulated cells. For each condition, n≥4. Differences between controls and stimulated cells were—if not otherwise noted—statistically significant (p<0.05).

In another set of experiments, cells from FcγRIII-deficient mice were tested for responsiveness to LPS. Peritoneal PMNs and macrophages from Wt and FcγRIII^−/−^ were incubated with LPS and opsonized zymosan (as a positive control) under the same conditions described above and supernatant fluids were analyzed for IL-6 and TNFα levels by ELISA. As shown in Figure 3, phagocytes from FcγRIII^+/+^ and FcγRIII^−/−^ mice robustly produced cytokines when incubated with LPS, opsonized zymosan or IgGIC. There was no difference in cytokine secretion between the FcγRIII^+/+^ and FcγRIII^−/−^ cells, except for LPS-induced TNFα release by FcγRIII^−/−^ PMNs, which was lower as compared to FcγRIII^+/+^ PMNs, but significantly elevated above baseline levels. As expected, FcγRIII^+/+^ macrophages robustly released IL-6 and TNFα into supernatant fluids when stimulated with IgGIC, whereas macrophages from FcγRIII^−/−^ mice were unresponsive to IgGIC (Figure 3C,D).

These results suggest that FcγRIII-deficient phagocytes can respond to LPS and that FcγRIII is not required for direct TLR4 signaling, while FcγRIII is essential for the mediation of IgGIC-induced responses.

### Phosphorylation of FcR γ-Subunit Requires the Integrity of TLR4

**Figure 4:**
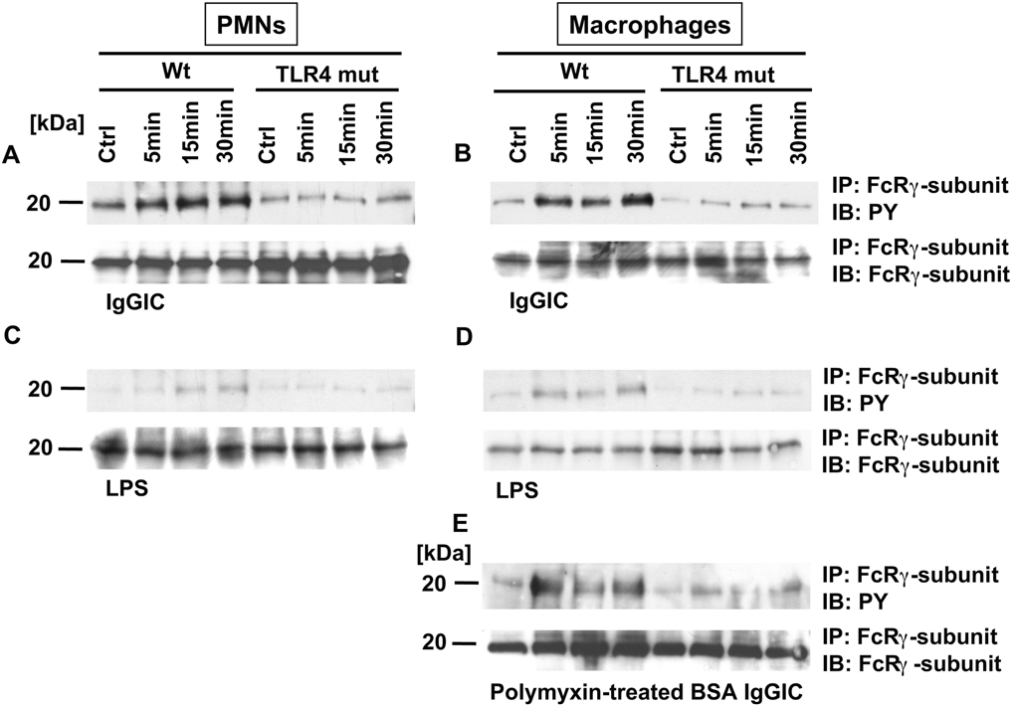
Western blot analysis for tyrosine-phosphorylated (PY) FcRγ-subunit of PMN or macrophage lysates after *in vitro* incubation. (A,B) 3×10^6^ cells/ml from either Wt or TLR4 mut mice were incubated for 5, 15, and 30 min with IgG immune complexes (IgGIC; 100 µg/ml). (C,D) The same protocol was used for stimulation with LPS (20 ng/ml). (E) Lysates from either Wt or TLR4 mut mice that were incubated with polymyxin-treated BSA immune complexes (100 µg/ml) under the same conditions as described above. Corresponding loading controls are displayed in the lower panels.

After binding of LPS, TLR4 engages intracellular signaling pathways via the adaptor molecules MyD88 and TRIF [27]. In the case of FcγR-immune-complex interaction, intracellular pathways are activated by tyrosine phosphorylation of the FcRγ-subunit ITAM region [8],[28]. This subunit is known to be the common adaptor of FcγRI, FcγRIII and FcεRI [29],[30], the first two being essential for development of IgGIC induced acute lung injury [31]. In order to evaluate the mechanism behind the impaired response of TLR4 mut cells to IgGIC, tyrosine phosphorylation of the FcRγ-subunit was investigated *in vitro*. When peritoneal PMNs (Figure 4A) or macrophages (Figure 4B) from Wt mice were exposed to IgGIC, rapid tyrosine phosphorylation (PY) of the FcRγ-subunit occurred over the first 30 min, as indicated by robust bands in the Western blots. In striking contrast, phosphorylation of the FcRγ-subunit failed to occur when TLR4 mut cells were used. Here, the intensity of the bands was comparable to those in non-stimulated cells (Figure 4A,B). When LPS was used as a stimulus (Figure 4C,D), slight phosphorylation of the FcRγ-subunit occurred in Wt cells (but not in TLR4 mut cells), indicating that TLR4 has little ability to activate the FcRγ-subunit as an intracellular signaling event (Figure 4C,D). Furthermore, the above mentioned results were confirmed in macrophages by using polymyxin-treated BSA IgGIC for stimulation under the same conditions in order to exclude LPS contamination of the reagents (Figure 4E). Collectively, these data suggest that the integrity of TLR4 seems to be required for a proper function of FcγR activation via phosphorylation of the FcRγ-subunit, further suggesting communication between the TLR4 and FcγR signaling pathways.

### Acute Lung Injury in Wt, TLR4 Mutant, and TLR4^−/−^ Mice

**Figure 5:**
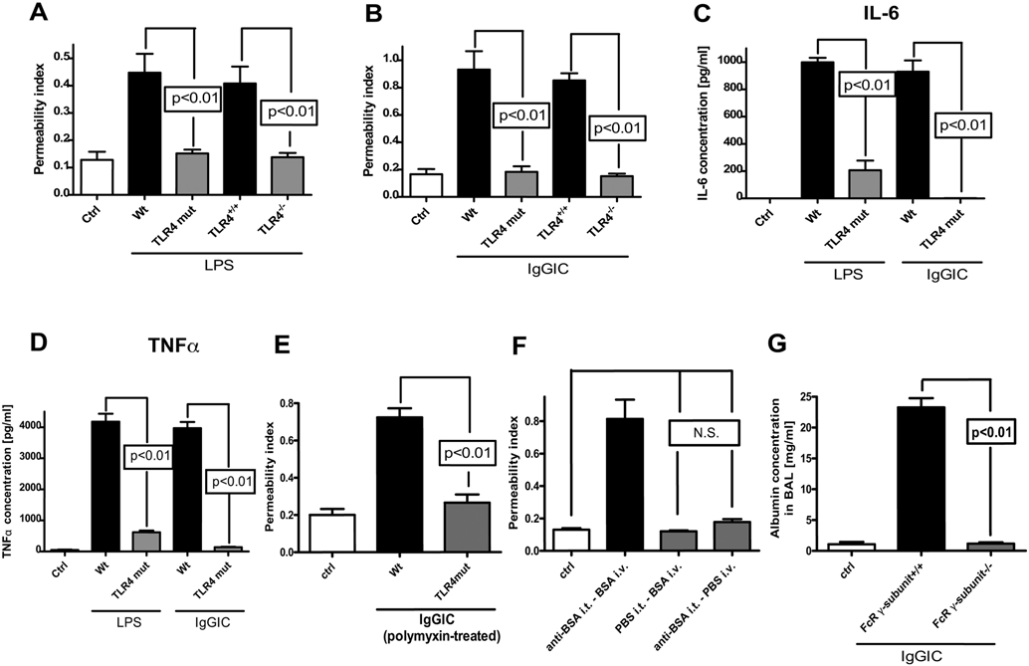
Parameters of acute lung injury in Wt and TLR4 mut mice. (A) Lung injury (as measured by leak of ^125^I-BSA into lung) in Wt, TLR4 mut, TLR4^+/+^, and TLR4^−/−^ mice receiving LPS intratracheally. (B) Permeability indices in Wt, TLR4 mut, TLR4^+/+^, and TLR4^−/−^ mice after intrapulmonary immune complex formation following administration of BSA (i.v.) and anti-BSA IgG (i.t.). (C) IL-6 levels in BAL fluids after IgG immune complex (IgGIC)- or LPS-induced lung injury using Wt and TLR4 mut mice. (D) TNFα in BAL fluids from the same mice described in frame (C). For each bar, n≥5. (E) Lung injury induced by IgG immune complexes (IgGIC) in Wt and TLR4 mut mice after endotoxin removal by polymyxin. (F) Lung permeability after intratracheal (i.t.) administration of anti-BSA IgG and intravenous (i.v.) injection of BSA, PBS i.t., and BSA i.v. or anti-BSA i.t. and PBS i.v. (G) IgGIC-induced lung injury in FcRγ-subunit^−/−^ mice in comparison to Wt mice (FcRγ-subunit^+/+^). For each bar, n≥5.

Using the LPS and IgGIC models of ALI, Wt, TLR4 mut, TLR4^+/+^ and TLR4^−/−^ mice were evaluated for responses following lung deposition of IgGIC or LPS. While FcγRs play a key role in the IgG immune complex (IgGIC) model of ALI [31],[32], TLR4 is critical for the development of lung injury in the LPS model [33],[34],[35]. As indicated in Figure 5A, LPS-induced lung injury, as defined by the permeability index (leak of plasma albumin into the extravascular lung compartment), showed a 4-fold increase in Wt mice (compared to controls, ctrl) and remained at the control level in LPS-challenged TLR4 mut mice. In the case of IgGIC (Figure 5B), the permeability index rose 5-fold above control (basal) levels in Wt mice. However, TLR4 mut mice unexpectedly showed no evidence of injury after deposition of IgGIC (Figure 5B). TLR4^−/−^ mice behaved similar to TLR4 mut mice in terms of lung injury, with virtually no lung injury in response to deposition of either LPS or IgGIC (Figure 5A,B). When IL-6 levels were measured in bronchoalveolar lavage (BAL) fluids, LPS and IgGIC induced high levels of IL-6 in Wt mice and very low levels in TLR4 mut mice (Figure 5C). Similar patterns were found for TNFα levels (Figure 5D).

Similarly, induction of ALI by intrapulmonary deposition of polymyxin-treated BSA IgGIC in Wt and TLR4 mut mice (Figure 5E) revealed no difference to the results displayed in Figure 5B; when polymyxin-treated reagents were administered for intrapulmonary IgGIC formation lung permeability rose 3.5 fold in Wt mice whereas mice TLR4 mut mice did not show a significant increase. Thus, these findings support the conclusion that lung injury induction by IgGICs is not linked to contamination of the reagents with endotoxin. In addition, reagents that were used for the formation of IgGIC were administered separately *in vivo* at the same concentration as they were used in combination for intrapulmonary IgGIC deposition (Figure 5F). When BSA was injected intravenously, followed by intratracheal PBS injection lung permeability was not different from control mice. Similarly, intratracheal injection of anti-BSA and subsequent intravenous DPBS injection (containing a trace amount of I^125^-labelled BSA) did not result in increased lung permeability. In striking contrast, the combination of anti-BSA (i.t.) and by BSA (i.v.) injection lead to the development of acute lung injury, as also shown in Figure 5B and 5E. These data indicate that the development of lung injury in the IgG model is dependent on the *in vivo* formation of immune complexes and may not be explained by putative LPS contamination of the reagents since their separate, independent administration failed to increase lung permeability. Finally, IgGIC lung injury was induced in FcR γ-subunit-deficient mice, which do not express FcγRI and FcγRIII on the surface of PMNs and macrophages [36]. In contrast to Wt mice (FcR γ-subunit^+/+^), FcR γ-subunit^−/−^ mice did not develop acute lung injury after intrapulmonary IgGIC deposition, as determined by lung permeability (Figure 5G). These findings suggest that the IgGIC-induced lung injury using anti-BSA and BSA is strictly dependent on the FcγR-mediated signalling, and not on LPS-induced activation of TLR4. However, the caveat remains that there is always a concern about LPS contamination in the context of sensitive assays and *in vivo* responses. In particular, the possibility that LPS was present at concentrations below the detection limit of the available assays, which would not result in any *in vivo* (and *in vitro*) responses alone, but would be responsible for putative synergistic effects and an augmentation of IgGIC-induced inflammatory responses cannot be entirely excluded.

### Expression of FcγRIII, FcRγ-Subunit, and C5aR in Wt and TLR4 Mutant Mice

**Figure 6:**
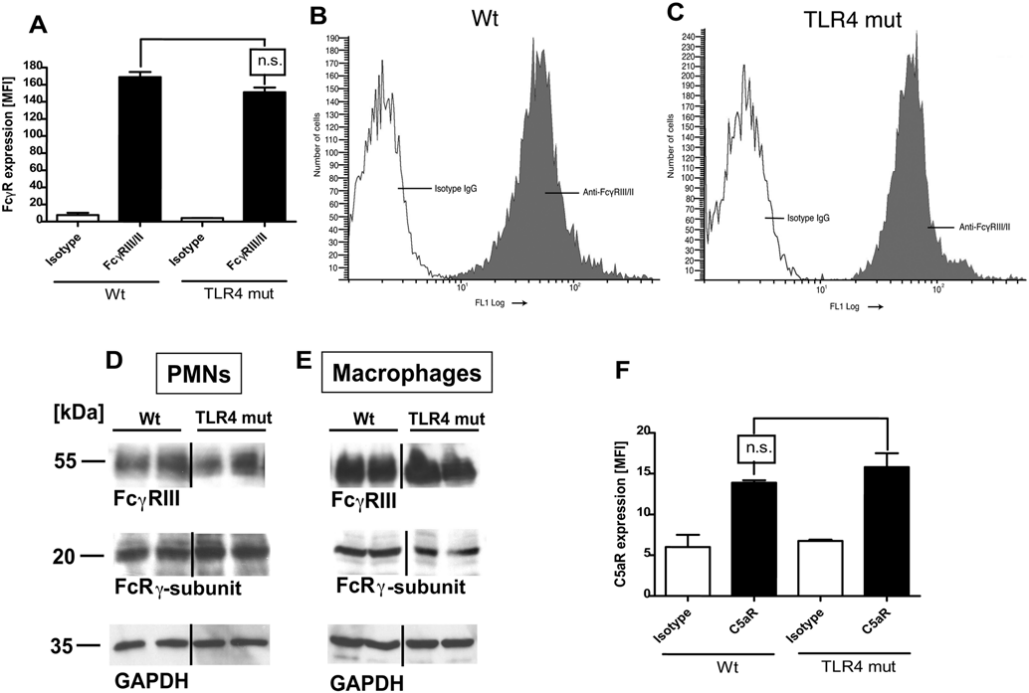
Expression levels of FcγRII/III, FcRγ-subunit, and C5aR on phagocytes from Wt and TLR4 mut mice. (A) Summary of flow cytometry analyses of FcγRII/III expression on blood PMNs. (B,C) Original flow cytometry results for FcγRII/III expression on the surface of PMNs from Wt (B) or TLR4 mut (C) mice. (D,E) Analysis of the expression of FcγRIII (upper bands) and FcRγ-subunit (middle bands) in cell lysates [(D), PMNs; (E), macrophages] from Wt or TLR4 mut mice by Western blotting. The lower bands represent the analysis for GAPDH as loading controls. (F) Surface expression of C5aR protein on PMNs from Wt or TLR4 mut mice as assessed by flow cytometry. MFI, mean fluorescence intensity. Studies were done in three separate and independent experiments, with each sample run in duplicates.

It is well established that engagement of FcγRIII with IgGIC as well as activation of the complement system with generation of C5a and its interaction with C5aR play crucial roles in the pathogenesis of IgGIC-induced ALI [31],[37],[38]. Therefore, elicited peritoneal PMNs were evaluated by flow cytometry for surface expression of FcγRII/III and C5aR protein. As shown in Figure 6A,F, the levels of each receptor on the surface of PMNs were the same in Wt versus TLR4 mut cells. The original flow cytometry data of FcγRII/III expression on Wt and TLR4 mut PMNs are displayed in Figure 6B,C. In addition, the total content of FcγRIII and FcRγ-subunit in cell lysates from Wt and TLR4 mut PMN (Figure 6D) and macrophages (Figure 6E) were analyzed by Western blotting. In accordance with the flow cytometry results (Figure 6A,B), unstimulated phagocytes from both mouse strains expressed the same levels of FcγRII/III and FcRγ-subunit. The analysis for the house keeping protein GAPDH (lower bands) indicates equal loading of the cell lysates. Thus, the inability of TLR4 mut mice to respond to IgGIC or LPS is not associated with reduced surface content of FcγR protein on PMNs, consistent with the findings that there is cross-talk between FcγR and TLR4 signaling pathways such that downstream production of IL-6 and TNFα upon IgGIC stimulation requires participation of both pathways. Collectively, these data indicate that TLR4 is required for proper FcγRIII functions.

## Discussion

The mechanisms by which the recognition of pathogens leads to host responses are inadequately understood. The modulation of immune responses is inter alia mediated by cell surface receptors that are associated with signaling molecules that contain ITAMs (immunoreceptor tyrosine-based activation motifs), TREMs (triggering receptors expressed on myeloid cells) and OSCARs (human osteoclast-associated receptors) [1]. Intracellular signaling after TLR4 activation is mediated through the adaptor proteins, MyD88 and TRIF, whereas FcγRI and FcγRIII both contain the FcRγ-subunit, which is phosphorylated at tyrosine residues by Src and Syk kinases upon FcγR activation [28],[30],[39],[40]. Interestingly, ligation of FcRγ-subunit containing FcRs results in inhibition of IL-12 production by monocytes in response to TLR ligands [41]. The specificity of IL-12 downregulation appears to be based on inhibition at the transcription level [41]. Moreover, TLRs are considered to control activation of acquired immunity [14], supporting the hypothesis for an instructive role of innate immunity in adaptive immune responses [15].

In the present study, we describe that TLR4 and FcγRIII associate, possibly by heterodimerization, following stimulation with IgGIC *in vitro* (Figure 1). Binding of IgGICs to the extracellular domain of FcγRs causes clustering of these receptors, followed by phosphorylation of tyrosine residues within the ITAM region, and subsequent activation of intracellular signaling cascades [28],[30],[40]. TLR signaling is initiated by dimerization of TLRs, which can form homo- or heterodimers [42]. Previously, it has been suggested that TLR4 co-associates with FcγRIII after activation of human monocytes [43]. Based on our findings, it is possible that TLR4 and FcγRIII multimerize into clusters following stimulation by LPS or IgGIC, a mechanism known as capping [44], which is required for engagement of intracellular signaling pathways. TLR4 may represent the central component for such signaling or “docking platforms” [45] and interconnect intracellular signaling pathways via association to adaptor proteins. As demonstrated in the present study, dysfunction of TLR4 results in impaired signaling in FcγRIII pathways (Figure 4).

The mutation that is responsible for the endotoxin tolerance of C3H/HeJ mice has recently been demonstrated to cause suppressed tyrosine phosphorylation by Src tyrosine kinases (Lyn) in the toll-IL-1 resistance (TIR) domain of TLR4, resulting in signaling-incompetence [45]. Altered or suppressed TLR4 tyrosine phosphorylation correlated with impaired MyD88 association and suppressed IRAK-1 activation [45]. In addition, our data suggest that this mutation in the TLR gene not only hinders phosphorylation of its own TIR domain but also blocks the tyrosine phosphorylation of the ITAM-containing FcRγ-subunit, the consequence of which ultimately leads to impaired signaling after engagement of FcγRIII.

In the LPS model of acute lung injury, TLR4 mut or TLR4^−/−^ mice were, as expected, highly protected from the development of tissue damage in the LPS-induced model of acute lung injury (Figure 5). It is well established that mice with mutation in the TLR4 gene or genetic deficiency of TLR4 are non-responsive to LPS [4], including LPS-mediated lung injury [33],[34],[35]. In the present study, TLR4 mut and TLR4^−/−^ mouse strains unexpectedly also showed greatly attenuated susceptibility to IgGIC-induced lung injury (Figure 5). For this model, it is known that, besides complement activation, FcγRs are critical for initiation and development of IgGIC alveolitis [31],[32], particularly through engagement and activation of ITAM-containing FcγRs (FcγRI and FcγRIII) [31]. In accordance, mice with targeted disruption of the FcRγ-subunit showed an impaired inflammatory response in the reverse passive Arthus reaction [46]. In our study, TLR4 mut mice not only were resistant to lung injury, but also failed to locally release cytokines *in vivo* after intrapulmonary IgGIC deposition, as indicated by baseline levels of IL-6 and TNFα in BAL fluids (Figure 5). In companion experiments, *in vitro* exposure of TLR4 mut phagocytes to IgGIC resulted in complete suppression of proinflammatory cytokines (TNFα, IL-6) in comparison to phagocytes from Wt mice (Figure 2). Furthermore, TLR4 mut cells showed impaired tyrosine phosphorylation of the FcRγ-subunit when exposed to IgGIC, in striking contrast to Wt cells (Figure 4). The fact that TLR4 mut PMNs and macrophages responded with cytokine release when incubated with opsonized zymosan particles or with Pam3Cys (Figure 3) indicates that 1.) the mutation in the TLR4 gene does not lead to a global impairment of the cellular inflammatory/immune response and 2.) the intracellular signaling pathways are intact since other TLRs (such as TLR2 and TLR6), which share common pathways, could be activated *in vitro*. On the other hand, phagocytes from FcγRIII-deficient mice are fully responsive to LPS (Figure 3), suggesting that TLR4 signaling does not depend on the functional integrity of FcγRIII, whereas TLR4 is required for FcγRIII signaling.

Especially in the field of immunology, there is an increasing number of reports describing effects of receptor interactions. Examples include a previous study suggesting cross-talk between IFN-gamma and IFN-alpha receptors with signaling pathways [47]. In brief, signalling by IFN-gamma was shown to depend on the IFN-alpha/beta receptor components. A more recent publication describes that signalling triggered by NKG2D and DAP10 is coupled to the interleukin 15 receptor signalling pathway, suggesting that coupling of activating receptors to other receptor systems may regulate cell type-specific signaling events [48]. In the case of innate immunity, it has been proposed several times that there is a link between TLR4 and the complement system, especially to the C5a signalling pathway, which can negatively regulate TLR4-induced responses [49],[50]. Under physiological conditions, receptor interactions and cross-talk between signalling pathways might represent important regulatory mechanisms of the immune system to provide distinct but fine-tuned responses. In the case of TLR4 and FcγRIII, cross-talk may provide an optimal and rapid response against invading microorganisms by mediating an interplay between adaptive and innate immunity. However, in certain conditions, such as systemic inflammation (sepsis) or autoimmune diseases that are characterized by a loss of inhibitory action or uncontrolled activation of signalling pathways, a loss of control over otherwise carefully orchestrated receptor interactions can become instruments of harm.

Taken together, the present findings strongly suggest that (*i*) there is a direct link between TLR4 and FcγR pathways, (*ii*) phosphorylation of tyrosine residues in the ITAM-containing FcRγ-subunit requires the presence and integrity of TLR4 during cellular activation after binding of IgGICs to FcγRs, and (*iii*) presence of IgGICs results in an association between TLR4 and FcγRIII (CD16) on phagocytic cells. These data imply that innate and adaptive immunity are closely connected at the receptor level and post receptor signaling pathways, which might have ramifications for a variety of inflammatory conditions, such as IgGIC-mediated autoimmune diseases (rheumatoid arthritis or glomerulonephritis), ischemia/perfusion injury, trauma or systemic inflammation (sepsis), etc.

## Materials and Methods

### Animals

Adult male (22–25 g) specific pathogen-free C3H/OuJ (Wt) and C3H/HeJ (TLR4 mut) mice with a missense mutation in the TLR4-gene were used in these studies [4]. In addition, lung injury was employed in mice lacking the genes for TLR4 (TLR4^−/−^; C57BL/10ScCr) and the corresponding wild-type mice (TLR4^+/+^; C57BL/ScSn) [4]. In some *in vitro* experiments, cells from FcγRIII-deficient (FcγRIII^−/−^; B6.129P2-*Fcgr3^tm1Sjv^*/J), FcR γ-subunit-deficient (FcRγ-subunit^−/−^; B6.129P2-*Fcer1g^tm1Rav^*N12) and appropriate Wt mice (C57BL/6) were used [51].

### Ethics Statement

All studies were performed in accordance with the University of Michigan Committee on Use and Care of Animals.

### *In Vitro* Incubation of Peritoneal PMNs and Macrophages

Mouse peritoneal leukocytes were harvested 5 h (PMNs) or 5 days (macrophages) after intraperitoneal injection of thioglycolate into untreated Wt and TLR4 mut mice by peritoneal lavage with PBS. 3×10^6^ cells / sample were incubated in HBSS for up to 4 h at 37°C in the presence of LPS (20 ng/ml; serotype O111:B4; Sigma, St. Louis, MO), BSA IgG immune complexes (IgGIC, 100 µg/ml; MP Biomedicals), polymyxin-treated BSA IgG immune complexes (p.-t. BSA IC, 100 µg/ml), peroxidase/anti-peroxidase IgG immune complexes (PAP IC, 100 µg/ml; MP Biomedicals), opsonized zymosan particles (300 µg/ml; Sigma) or Pam3Cys (1 µg/ml; InvivoGen). After incubation, supernatant fluids were collected for assessment of cytokines by ELISA and pellets were lysed with RIPA buffer (Upstate) for immunoprecipitation analyses.

### Immunoprecipitation and Western Blotting

After incubation of peritoneal PMNs or macrophages with either IgG immune complexes (100 µg/ml; prepared as described elsewhere [52] or LPS (20 ng/ml) for 5 to 30 min, supernatant fluids were removed and pellets were lysed with 1X RIPA buffer containing Vanedate and protease inhibitors (Roche Diagnostics). Protein concentrations were determined in cell lysates using BCA protein assay (Pierce). Equal protein amounts of supernatants were then incubated overnight with preblocked protein A and G beads (Santa Cruz) in the presence of anti-FcRγ-subunit IgG (Upstate) or anti-TLR4 IgG(Santa Cruz), respectively. Reverse direction immunoprecipitation included anti-FcγRIII IgG (Santa Cruz).

After centrifugation, pellets were resuspended in Laemmli sample buffer (Biorad) followed by boiling of the samples. After a final spin step, supernatant fluids were electrophoretically separated under reducing conditions in SDS-PAGE and transferred onto PVDF membrane. The membrane was blocked in 5% bovine milk in TBST and then probed for TLR4 or FcγRIII using polyclonal anti-TLR4 IgG (1 µg/ml, Santa Cruz) or monoclonal anti-FcγRII/III IgG (1 µg/ml; clone 2.4G2; BD Pharmingen). Alternatively, membranes containing the samples co-immunoprecipitated with anti-FcRγ-subunit IgG were incubated with anti-phospho-tyrosine monoclonal antibody (1 µg/ml; clone 4G10, Upstate). As secondary antibodies, HRP-conjugated donkey anti-goat IgG (1∶80,000; Jackson Immunoresearch), HRP-conjugated goat anti-rat IgG (1∶10,000; Amersham) HRP-conjugated donkey anti-rabbit IgG (1∶10,000; Amersham) and HRP-conjugated sheep anti-mouse IgG (1∶20,000; Amersham) were added and the blot was developed using ECL-procedure (Amersham).

### ELISA for Mouse IL-6, TNFα

For measurement of IL-6 and TNFα in BAL fluids and supernatant fluids after *in vitro* incubation of mouse PMNs and macrophages, commercially available ELISA-kits (“Duo set”, R&D Systems) were used according to the manufacturer's protocol.

### Immune Complex Lung Injury

To induce IgGIC lung injury, tracheae of mice were surgically exposed and 125 µg rabbit anti-BSA IgG (MP Biomedicals) was administered using a 30 gauge needle (volume of 42 µl/mouse) followed by intravenous injection of BSA (500 µg; Sigma). For determination of the permeability index as a quantitative marker for vascular leakage, ^125^I-labelled bovine serum albumin (1 µCi ^125^I-BSA/mouse) was injected intravenously. After the development of acute lung injury, the pulmonary vasculature was flushed with 2.0 ml PBS. The amount of lung radioactivity was then measured as a ratio of radioactivity present in 100 µl blood recovered from the inferior vena cava at the time of animal euthanasia and that in lung. For bronchoalveolar lavage retrieval, lung injury was performed as described above, but without the intravenous injection of ^125^I-BSA. The airways were flushed with 0.8 ml ice cold PBS using a blunt 20 gauge needle and BAL fluids were recovered for further studies.

### LPS Lung Injury

50 µg LPS from *E.coli* (serotype O111:B4; Sigma) were given intratracheally (volume of 42 µl/mouse). When lung permeability was measured, a trace amount of ^125^I-BSA was injected intravenously, as described above. The permeability index was determined and BAL fluids were collected as described for the IgGIC model.

### Detection of Possible LPS Contamination

Reagents other than LPS, such as DPBS, BSA, anti-BSA IgG that were used for the *in vivo* and *in vitro* experiments were tested for LPS-contamination. For quantification of LPS content, samples were conducted in Limulus Amebocyte Lysate Kinetic-QCL assay (Cambrex) according to the manufacturer's protocol and as described elsewhere [53]. In addition, reagents used for immune complex formation (DPBS, BSA, anti-BSA IgG) were subjected to endotoxin removal (Pierce) prior to induction of lung injury or preparation of immune complexes used stimulation of phagocytes *in vitro*.

### Analysis of FcγR and C5aR on PMNs

Flow cytometric analysis was conducted after whole blood collection of untreated wild-type and TLR4 mut mice in a citrate-containing syringe. Rabbit anti-mouse C5aR serum (1∶10 dilution; Lampire) was incubated with mouse whole blood. Non-specific rabbit serum (Jackson Immunoresearch) was added to control samples in equal amounts. For detection of FcγR on PMNs, mouse whole blood was either incubated with 1 µg monoclonal anti-FcγRII/III IgG (clone 2.4G2; BD Pharmingen) or with the appropriate isotype IgG control (Jackson Immunoresearch). After washing, cells were suspended in Phycoerythrin (PE)-labeled anti-rabbit IgG (Invitrogen) diluted 1∶200 in staining buffer and incubated at room temperature for 45 min. Erythrocytes were lysed by addition of 1× FACS lysing solution (BD Pharmingen) for 10 min. After washing, the leukocytes were resuspended in a 1%-paraformaldehyde fixing solution and analyzed on a flow cytometer (BD Pharmingen).

### Statistical Analysis

All values were expressed as mean±SEM. Data sets were analyzed by one-way analysis of variance (ANOVA); differences in mean values among experimental groups were then compared using Tukey multiple comparison test. Results were considered statistically significant when *P*<0.05.

## Supporting Information

Figure S1 Cytokine response of PMNs and macrophages to Zymosan and Pam3Cys. *In vitro* cytokine responses to non-TLR4 agonists of elicited peritoneal phagocytes from Wt or TLR4 mut mice. PMNs (A–D) and macrophages (E–H) (3×10^6^ cells/ml) were incubated (for 4 hr) with serum-opsonized zymosan particles (300 µg/ml) or Pam3Cys (1 µg/ml). Ctrl = control levels of non-stimulated cells. For each condition n≥3. Differences between controls and stimulated cells were found to be statistically significant (p<0.05).
(0.50 MB EPS)
